# Supplementary material for: Association between cardiac autonomic regulation, visceral adipose tissue, cardiorespiratory fitness and ambient air pollution: 4HAIE study (Program–4)
Source: PLoS One. 2024 Dec 23;19(12):e0315767. doi: 10.1371/journal.pone.0315767 (PMC11666063; doi:10.1371/journal.pone.0315767)
Supplement: S1 Table — VO2peak−maximal aerobic power, rMSSD ‐ the root mean square value of the successive differences of the normal RR intervals, VAT–visceral adipose tissue. Data are expressed as mean ± SD (or median ±IQR). (DOCX) [file pone.0315767.s001.docx]

| Participants (female/male) | 1036 (487/549) |
| --- | --- |
| Moravian-Silesian Region/ South Bohemia Region | 569/467 (55%/45%) |
| Age | 38.1 (12.4) |
| Height (cm) | 174.6 (9.0) |
| Body Mass (kg) | 75.2 (14.3) |
| rMSSD (ms) | *48.5 (37.4)* |
| $\dot{V}$O_2peak_ (ml/min/kg) | 41.6 (10.3) |
| VAT_area_ (cm^2^) | *81.6 (46.2)* |
| Education level 1 *(basic, unfinished or apprentice)* | 135 (14%) |
| Education level 2 *(secondary school diploma)* | 420 (40%) |
| Education level 3 *(higher vocational, university and higher)* | 481 (46%) |

**Table S1.** Characteristics of the participants (data before logarithmisation)

Legend: VO_2peak_ – maximal aerobic power, rMSSD - the root mean square value of the successive differences of the normal RR intervals, VAT – visceral adipose tissue. Data are expressed as mean ± SD (or *median ±IQR).*
