# Supplementary material for: Association between cardiac autonomic regulation, visceral adipose tissue, cardiorespiratory fitness and ambient air pollution: 4HAIE study (Program–4)
Source: PLoS One. 2024 Dec 23;19(12):e0315767. doi: 10.1371/journal.pone.0315767 (PMC11666063; doi:10.1371/journal.pone.0315767)
Supplement: S2 Table — MSR–Moravian-Silesian Region, SBR–South Bohemia Region, EU–European Union, PM–Particulate matter, NO2 –nitrogen dioxide. Data are expressed as mean ± SD. (DOCX) [file pone.0315767.s002.docx]

**Table S2. –** Differences between MSR and SBR as long-term averages of the exposure to air pollutants in 2000 - 2017 (Machaczka et al., 2023).

|  | PM_10_  (μg/m^3^) | PM_2.5_  (μg/m^3^) | NO_2_  (μg/m^3^) | Benzene (μg/m^3^) | Benzo[a]pyrene (ng/m^3^) |
| --- | --- | --- | --- | --- | --- |
| MSR | 45.6 (2.4) | 34.9 (1.9) | 18.1 (1.0) | 2.1 (0.1) | 2.6 (0.1) |
| SBR | 24.9 (1.7) | 19.4 (1.3) | 13.3 (1.0) | 0.8 (0) | 0.4 (0) |
| EU air quality standards | 40 | 20 | 40 | 5 | 1 |

**Legends:** MSR – Moravian-Silesian Region, SBR – South Bohemia Region, EU – European Union, PM – Particulate matter, NO_2_ – nitrogen dioxide. Data are expressed as mean ± SD.
