# Supplementary material for: Association between cardiac autonomic regulation, visceral adipose tissue, cardiorespiratory fitness and ambient air pollution: 4HAIE study (Program–4)
Source: PLoS One. 2024 Dec 23;19(12):e0315767. doi: 10.1371/journal.pone.0315767 (PMC11666063; doi:10.1371/journal.pone.0315767)
Supplement: Attachment — Submitted filename: Considerations.docx [file pone.0315767.s003.docx]

Methodological Considerations:

1. What methodology was employed to assess individual physical activity levels? Was self-reporting utilized? How was strength training accounted for in the analysis?
2. Were ambient air pollution levels measured concurrently with HRV assessments? If not, how was temporal variability in air quality addressed?
3. The study design appears to have omitted consideration of physical activity intensity. What was the rationale for this exclusion, and how might it impact the interpretation of results?
4. The broad exclusion criteria may introduce selection bias. What measures were implemented to mitigate this potential confounding factor?

Results Interpretation and Discussion:
5. The discrepancy between this study's findings and previous literature warrants further elucidation. A more comprehensive comparative analysis of methodologies and populations might shed light on these inconsistencies.

6. The manuscript would benefit from more rigorous scientific language and terminology throughout.

Study Limitations:
7. The cross-sectional design inherently limits causal inference. This limitation should be explicitly acknowledged and its implications for result interpretation discussed.

8. The use of regional classifications as a proxy for air pollution exposure, rather than direct measurements, introduces potential measurement bias. How might this affect the validity of the pollution-related findings?
9. The HRV measurement protocol lacks sufficient detail. Factors such as measurement duration, diurnal timing, and environmental conditions can significantly influence HRV parameters and should be thoroughly described.

Recommendations for Revision:
10. Provide a comprehensive description of the HRV measurement protocol in the methods section, including all relevant parameters and environmental controls.

11. Expand the discussion to address potential mechanisms underlying the observed lack of association between air pollution exposure and HRV. This should include a critical evaluation of the limitations inherent in using regional classifications as exposure proxies.
12. Consider conducting additional statistical analyses to explore potential interaction effects, particularly between CRF and air pollution exposure, which could reveal more nuanced relationships.

Conclusion:
This study contributes valuable data to the field of cardiac autonomic regulation and its relationship with various health-related factors in a large cohort. While the absence of an association between HRV and air pollution exposure is unexpected, the strong correlations with CRF and age provide important confirmatory evidence. With the suggested revisions and an expanded discussion, this manuscript has the potential to make a significant contribution to the literature on cardiac autonomic regulation and environmental health.
